# Protein Intake, IGF-1 Concentrations, and Growth in the Second Year of Life in Children Receiving Growing Up Milk – Lite (GUMLi) or Cow's Milk (CM) Intervention

**DOI:** 10.3389/fnut.2021.666228

**Published:** 2021-06-10

**Authors:** Amy L. Lovell, Tania Milne, Misa Matsuyama, Rebecca J. Hill, Peter S. W. Davies, Cameron C. Grant, Clare R. Wall

**Affiliations:** ^1^Department of Nutrition and Dietetics, Faculty of Medical and Health Sciences, University of Auckland, Auckland, New Zealand; ^2^Faculty of Medical and Health Sciences, University of Auckland, Auckland, New Zealand; ^3^Child Health Research Centre, University of Queensland, Brisbane, QLD, Australia; ^4^Department of Paediatrics: Child and Youth Health, University of Auckland, Auckland, New Zealand; ^5^Centre for Longitudinal Research He Ara ki Mua, University of Auckland, Auckland, New Zealand; ^6^General Paediatrics, Starship Children's Hospital, Auckland District Health Board, Auckland, New Zealand

**Keywords:** cow's milk, Young Child Formula, growing-up milk, young children, IGF-1, growth

## Abstract

The relationship of protein intake with insulin-like growth factor 1 (IGF-1) concentrations in well-nourished children during the second year of life is poorly understood. The aim of this study was to explore the effect of a reduced-protein Growing Up Milk Lite (GUMLi) or unfortified cow's milk (CM) on protein intake, growth, and plasma IGF-1 at 2 y. An exploratory analysis of a sub-sample of Auckland-based children (*n* = 79) in the GUMLi trial (a double-blind, randomised control trial, *N* = 160) completed in Auckland and Brisbane (2015–2017) was conducted. One-year old children were randomised to receive a reduced-protein GUMLi (1.7 g protein/100 mL) or a non-fortified CM (3.1 g protein/100 mL) for 12 months. Blood sampling and anthropometric measurements were made at 1 and 2 y. Diet was assessed using a validated food frequency questionnaire. Total protein intake (g/d) from all cow's milk sources was 4.6 g (95% CI: −6.7, −2.4; *p* < 0.005) lower in the GUMLi group after 12 months of the intervention, with a significant group-by-time interaction (*p* = 0.005). Length-for-age (LAZ) and weight-for-length (WLZ) *z*-scores did not differ between groups, however, mean body fat % (BF%) was 3.2% (95%CI: −6.2, −0.3; *p* = 0.032) lower in the GUMLi group at 2 y. There was no difference between the intervention groups in relation to IGF-1 and IGF-BP3 (*p* = 0.894 and 0.698, respectively), with no group-by-sex interaction. After combining the groups, IGF-1 concentration at 2 y was positively correlated with parameters of growth (all *p* < 0.05), total cow's milk intake (*p* = 0.032) after adjusting for sex, breastfeeding status, and gestation. Randomisation to a reduced protein GUMLi resulted in small reduction in %BF and lower total protein intakes but had no effect on growth. Plasma IGF-1 concentrations were independently associated with total protein intake from cow's milk at 2 y, highlighting a potential area of the diet to target when designing future protein-related nutrition interventions.

**Clinical Trial Registration:** Australian New Zealand Clinical Trials Registry number: ACTRN12614000918628. Date registered: 27/08/2014.

## Background

During the transition to a family-style diet, a child's protein intake increases significantly, often exceeding physiological demands and creating an imbalance between age-related protein requirements and growth velocity (1, 2). The World Health Organization has suggested lower protein intakes of ≤ 15% total daily energy intake (EI) as safe levels of protein intake for infants and young children (3). However, recent observational cohort studies of Australian (4), Belgian (5), and Irish children (6) have reported intakes of 2–3 times country-specific recommendations.

The Early Protein Hypothesis, first proposed by Rolland-Cachera et al. (7), states that high protein intake in excess of metabolic requirements, usually associated with formula during early infancy and cow's milk from 1 y of age, has been correlated with increased secretion of growth mediators insulin and insulin-like growth factor I (IGF-1) which enhances fat deposition and weight gain (7–12) alongside risk of obesity and adiposity associated disease (13). It is difficult to ascertain whether early life protein intake sets a precedent for continued high protein intake throughout childhood. However, the association between dietary protein intake exceeding 15% total EI during complementary feeding with increased linear growth, weight gain, and measures of adiposity such as BMI *z*-score during the first 2 y of life and beyond has been reported (9, 14–16).

With Dietary Guidelines recommending the inclusion of cow's milk from 1 y of age (17), milk continues to be a significant determinant of total energy and protein intake during early childhood (4, 5). Several studies have reported an association of %EI from cow's milk with secretion of IGF-1 and measures of adiposity in later childhood (18–23). Of note, a stronger growth-stimulating effect has been reported in children with compromised nutritional status compared to well-nourished children (21, 23, 24). Thus, it appears that protein quantity and quality (including source) combined with nutritional status are factors driving this obesogenic relationship.

The relationship between protein intake and stimulation of IGF-1 is an important consideration in the context of Growing Up Milks (GUM) or Young Child Formula (YCF), which are often a significant protein source in a child's diet (4, 5, 25) and are frequently given to children in higher-income countries (26, 27). The aim of this study was to explore the effect of a reduced-protein Growing Up Milk Lite (GUMLi) or unfortified cow's milk (CM), commencing at 1 y on total protein intake, plasma IGF-1 and IGFBP-3 concentrations and growth at 2 y in a sub-sample of Auckland-based children participating in the GUMLi randomised controlled trial.

## Methods/Design

### Study Design

**Figure 1:**
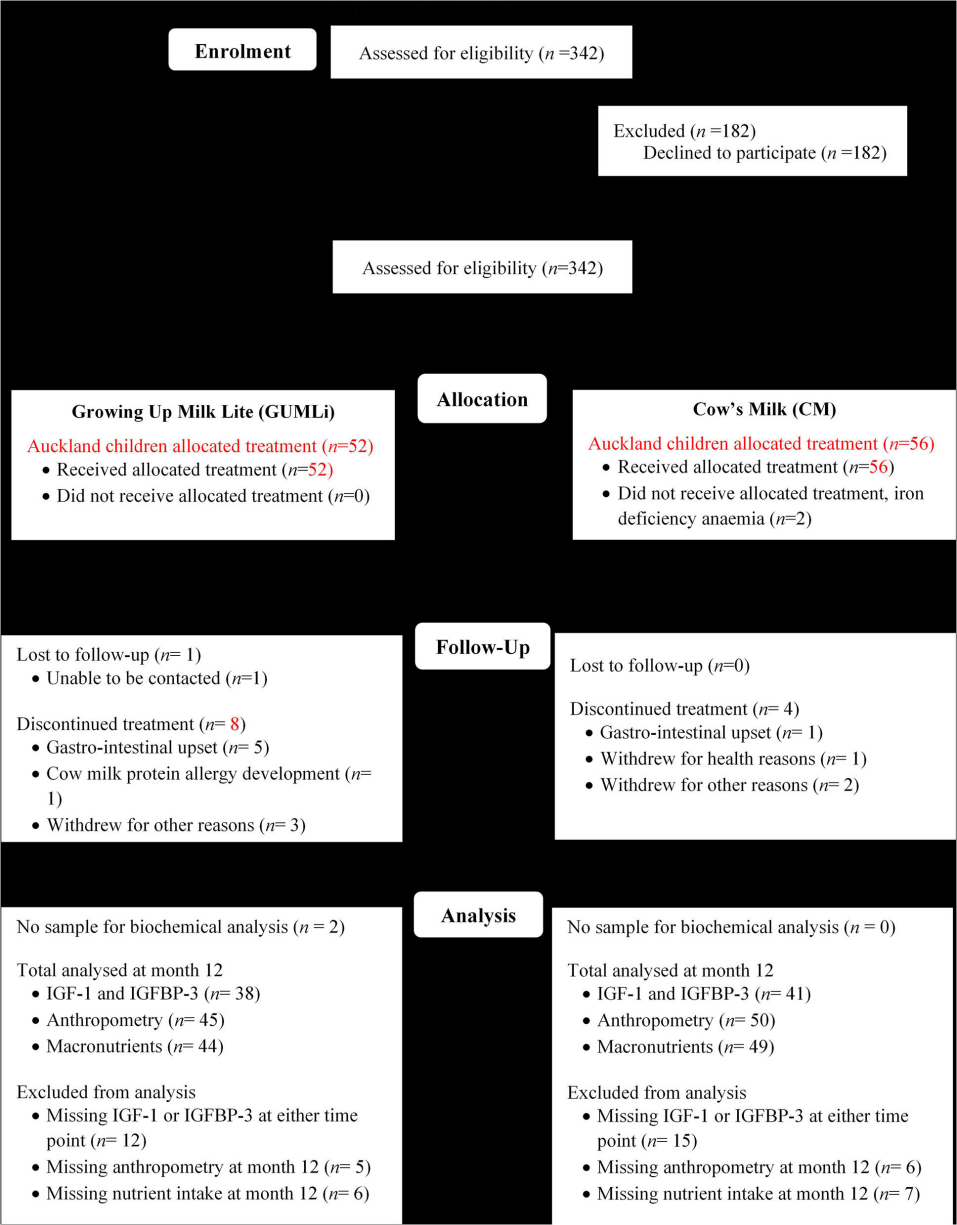
Modified CONSORT flow diagram showing Auckland GUMLi Trial participant randomisation, allocation, follow up and study analysis. CONSORT, CONsolidated Standards Of Reporting Trials; IGF-1, insulin-like growth factor 1; IGFBP-3, insulin-like growth factor binding protein 3.

This study represents an exploratory analysis of the GUMLi Trial in a sub-sample of Auckland-based children. The GUMLi trial was a multi-centre double-blind randomised, comparator-controlled trial designed to compare the effect on body composition at 2 y of a reduced protein GUM (GUMLi) fortified with iron, vitamin D, pre- and probiotics (synbiotics) vs. unfortified CM as part of a whole diet for 12 months (28). The trial was multisite, conducted in Auckland, New Zealand and Brisbane, Australia (2015–2017). Figure 1 shows the study outline. Ethical approvals were obtained from the Northern B Health and Disability Ethics Committee (HDEC) of the New Zealand Ministry of Health (HDEC reference number 14/NTB/152) and the University of Queensland Medical Research Ethics Committee (MREC) in Brisbane, Australia (reference number 2014001318). Written, informed consent to participate in the trial was provided by primary caregivers of all enrolled children.

### Participants

Healthy children, 1-year of age (±2 weeks) were recruited from Auckland (*n* = 108) and Brisbane (*n* = 52). Participants were randomised 1:1 to one of two treatment groups: GUMLi or whole pasteurised and homogenised, unfortified cow's milk (CM), both provided in powder form for a period of 12 months. Surplus plasma samples were available for the current analysis in a sub-sample of Auckland children only (*n* = 79) at baseline and month 12 of the intervention. No surplus samples were available from children enrolled in the Brisbane arm of the GUMLi trial.

### Study Intervention and Monitoring

The full study design is described in detail elsewhere (28). Briefly, participants were randomly assigned a unique numeric identifier on randomisation into the trial. Data were consolidated using Research Electronic Data Capture Software (REDCap) (29), a secure, password-protected web application, managed by the University of Queensland. The study milks were provided in powder form with instructions on reconstitution with water. The milks differed in the amount of cow's milk protein per 100 mL (22 and 12% of energy, CM and GUMLi, respectively) however, were energy-matched (245 kJ and 249 kJ/100 mL, CM and GUMLi, respectively). GUMLi was lower in total fat (supplied as milk fat with added long-chain polyunsaturated fatty acids), was fortified with micronutrients, including iron and vitamin D, and probiotics, and prebiotics (synbiotics). GUMLi had no added sucrose, dextrose, or flavours, however, had higher total carbohydrate content, attributed to higher lactose content. The whey:casein ratio in GUMLi was matched to CM at 80:20 (Supplementary Table 1). Participants were requested to consume at least 300 mL of the study milk per day. Adherence was measured at monthly intervals throughout the 12 month intervention and was defined as consumption of ≥300 mL study milk/d on 80% of the days within the previous month. Total adherence to the study protocol was calculated as the mean across the 12 month intervention period. Parents were not provided with any dietary advice during the intervention and continued breastfeeding was encouraged and supported throughout the trial. Participants that were consuming any formula prior to randomisation were asked to discontinue at baseline and only offer the study milk (CM or GUMLi) for the duration of the intervention. If children required additional milk each day beyond the 300 mL of intervention milk, parents were asked to offer whole cow's milk, as per the Dietary Guidelines for children >1 year of age (17).

### Dietary Intake

Dietary intake was assessed using the interviewer-administered Eating Assessment in Toddlers Food Frequency Questionnaire (EAT FFQ) at baseline and month 12 of the intervention (30, 31). The EAT FFQ is a previously validated, New Zealand-specific FFQ designed to describe dietary intake of children 1–2 y and has been validated and calibrated in the GUMLi population, following additions of food items and to determine the validity of providing an estimate of vitamin D intake from food (32). Intakes of energy (kcal/d) and protein (g/d) were calculated as previously described, using a custom written programme in MATLAB® (MathWorks Inc., United States) verified by hand calculations (33). Intake of cow's milk as a liquid was hypothesised to influence IGF-1 concentrations, therefore was a variable of interest. Cow's milk was defined as whole and skim milk, served as a drink or with/in food. Both the unfortified CM and intervention GUMLi were cow's milk-based products, therefore counted towards total daily cow's milk intake.

### Anthropometric Measurements

Standardised anthropometric measurements including weight (kg), recumbent length (cm), BMI (kg/m^2^), and body composition determined by a single frequency bioelectrical impedance (BIA) device (Bodystat 1500 MD) were performed by trained GUMLi study staff and have been described in detail elsewhere (28).

### Sample Collection and Analysis

A non-fasted 3 mL finger prick capillary sample was collected by an experienced paediatric phlebotomist at baseline and month 12 of the intervention. Samples were collected into a 0.25 mL EDTA tube (full blood count), two 0.5 mL serum separate tubes (c-reactive protein and iron studies) and a plain 0.5 mL tube for 25-hydroxyvitamin D concentration measurements. Within 2 h of collection, blood in the serum separator tubes was centrifuged for 10 min at 3,000 rpm to separate the serum from whole blood. Samples were analysed by the local pathology laboratory, LabTests Auckland (c-reactive protein and iron studies) as part of the trial secondary analyses (34). LabTests regularly participate in external quality assurance testing and accuracy and precision of all laboratory measurements were determined via analysis of appropriate standards and controls. After biochemical analysis of the trial secondary outcomes (iron and vitamin D status) (34), surplus serum samples were frozen and stored (−80°C) at LabTests, Auckland. These samples were transferred to the Liggins Institute, the University of Auckland, defrosted and concentrations of IGF-1 and IGFBP-3 measured using a commercially available enzyme-linked immunosorbent assay (ELISA) kit (human serum/plasma IGF-1 and IGFBP-3 ELISA, Mediagnost, Germany). All laboratory steps were performed as per the manufacturer's instructions without any modification. The intra- and inter-assay coefficients of variation were 2.2 and 2.4% for IGF-1, respectively and 3.7 and 11% for IGFBP-3, respectively. Sensitivity was 0.7 ng/mL.

### Statistical Methods

Baseline descriptive statistics, including participant and parental characteristics were summarised by treatment group. Categorical variables were described as frequencies and percentages, normally distributed continuous variables as means ± SD, and non-normal continuous data as medians and quartiles. IGF-1 and IGFBP-3 concentrations were not normally distributed and were log transformed and the geometric mean displayed. All statistical analyses were performed using SPSS, version 26 for Windows (SPSS Inc., Chicago, IL, USA). Data was tested for normality using Kolmogoroc-Smirnov and Shapiro-Wilk tests, with *p* > 0.05 for either test treated as normally distributed. All model assumptions were checked and statistical tests were two-sided with a significance level of *p* < 0.05. Baseline variables between the GUMLi and CM groups were compared using independent *t*-tests for parametric variables and Mann-Whitney for non-parametric variables. For categorical variables, chi-squared tests were used, or Fisher's exact test with small cell counts (<5).

Repeated-measures ANOVA were used to evaluate the main effect of time and group and their interactions with the dependent variables. Student's *t*-test was used as a *post-hoc* analysis to compare values between groups at each time point (paired) and over time (independent) and changes over time within each group (paired). ANCOVA regression models were used to investigate the effect of the study milks on anthropometric, dietary and biochemical variables after 12 months of the intervention, adjusting for baseline outcomes, and child sex. A hierarchical multiple linear regression was used to test associations between IGF-1 and variables (growth and nutrient intake), adjusting for confounders. Partial correlations were performed on the sub-sample to identify relationships between growth, biochemical and dietary intake variables, controlling for sex, whether the child was still breastfeeding at 2 y, and gestation due to their known relationships with IGF-1.

## Results

### Participant Characteristics

A total of 79 (73%) children from the Auckland centre of the GUMLi trial (full Auckland cohort *n* = 108) had biochemical samples available for IGF-1 and IGFBP-3 concentration analysis at both baseline and month 12 of the intervention (Figure 1). Baseline characteristics are summarised in Table 1. There were no significant group differences for baseline sociodemographic characteristics, apart from gestation, where 11% (*n* = 6) of the CM children were reported as being born <37 weeks gestation compared to 0% in the GUMLi intervention (*p* = 0.019). Three of the children born <37 weeks and randomised to the CM intervention were VLBW (*n* = 1) or low birth-weight (LBW, *n* = 2) and 2 children randomised to the GUMLi intervention were LBW. The remaining child with a gestation of <37 weeks was not considered low birth weight. Forty percent of the participants were receiving breastmilk at baseline. There were no group differences in anthropometric characteristics at baseline, and prior to randomisation total baseline energy and protein intakes did not differ between groups.

**Table 1:** Birth and baseline characteristics of included Auckland participants (*n* = 108).

**Characteristic**	**Study Group**		***p*-value**
	**CM (*n* = 56)**	**GUMLi(*n* = 52)**	
Sex, *n* (%)			0.190^*^
Boy	35 (63)	26 (50)	
Girl	21 (37)	26 (50)	
Gestation, *n* (%)			0.015^*^
Term	50 (89)	52 (100)	
<37 weeks	6 (11)	0 (0)	
Still breastfed at enrolment, *n* (%)			0.325^†^
Yes	25 (45)	18 (35)	
No	31 (55)	33 (64)	
*missing*		*1 (2)*	
Attends day care, *n* (%)			0.795^*^
Yes	32 (57)	31 (60)	
No	24 (43)	21 (40)	
Child anthropometric measurements			
Baseline BMI (kg/m^2^), mean ± SD	17.5 ± 1.3	17.3 ± 1.3	0.981^§^
WAZ, median (Q1,Q3)	0.5 (−0.3, 1.3)	0.6 (0.2, 1.2)	0.742^‡^
LAZ, median (Q1,Q3)	0.1 (−0.6, 0.9)	0.4 (−0.6, 1.4)	0.190^‡^
zBMI, mean ± SD	0.6 ± 0.9	0.4 ± 0.8	0.935^§^
zWeight-for-length, mean ± SD	0.6 ± 0.9	0.5 ± 0.8	0.684^§^
Body Fat (%), mean ± SD	23.4 ± 6.8	22.4 ± 6.8	0.775^§^
Nutrient intake			
Energy (kcal/day), mean ± SD	1336.4 ± 532.2	1254.1 ± 380.1	0.680^§^
Protein (g/day), median (Q1, Q3)	46.0 (36.6, 58.0)	50.3 (38.0, 60.7)	0.412^‡^
Protein (%EI), median (Q1, Q3)	15.5 (14.1, 17.5)	16.3 (14.5, 18.0)	0.480^‡^
Protein (g/kg/day), median (Q1, Q3)	4.8 (3.9, 5.9)	4.8 (4.0, 6.0)	0.591^‡^
Total milk intake^||^ (mL/day), median (Q1, Q3)	470.6 (68.8, 719.6)	497.9 (160.3, 645.8)	0.883^‡^
Serum biomarkers			
IGF-1 (ng/mL), mean^¶^ ± SD	77.3 ± 58.0	71.0 ± 66.1	0.324^‡^
IGFBP-3 (ng/mL), mean^¶^ ± SD	2709.6 ± 759.2	2678.4 ± 991.7	0.500^‡^

* *Chi-squared*.

† *Fisher's exact test performed due to <5 expected counts*.

‡ *Mann-Whitney for non-parametric variables*.

§ *Student t-test*.

|| *Includes whole CM, CM based formula, Toddler Milk/Young Child Formula*.

¶ *Geometric mean*.

*BMI, body mass index; CM, cow's milk; EI, energy intake; GUML, Growing Up Milk – Lite; IGF-1, insulin-like growth factor 1; IGFB-3, IGF binding protein 3; LAZ, length-for-age z-score; Q, quartile; WAZ, weight-for-age z-score; Z z-score*.

### Dietary Intake

Total energy (kcal/d) and absolute protein intakes (g/d) increased over time, however, they did not differ significantly between groups at baseline or month 12 of the intervention (Table 2). There was considerable variation in the mean intake of cow's milk (whole and skim) as a drink in addition to the 300 mL of intervention milks, demonstrated by large standard deviations in volume (mean (SD), CM 101.5 (100.0) mL/d and GUMLi 88.7 (107) mL/d). There were no differences in additional cow's milk intakes (whole and skim cow's milk as a drink) between intervention groups after 12 months of the intervention milks (*p* = 0.423). Energy-adjusted daily protein intake was 2.8 g/1,000 kcal (95% CI: −5.4, −0.1; *p* = 0.040) lower in the GUMLI group after adjusting for baseline protein intake and child sex. Total protein intake (g/d) from all cow's milk drink sources was 4.6 g (95% CI: −6.7, −2.4; *p* < 0.005) lower in the GUMLi group after 12 months of the intervention. Notably, percent total protein intake (%PI) from cow's milk was 6.9% (95%CI: −10.4, −3.4; *p* < 0.005) lower in the GUMLi group at month 12. This resulted in a 4% increase and 30% decrease in %PI from cow's milk at month 12 in the CM and GUMLi groups, respectively.

**Table 2:** Anthropometric, macronutrient, and biochemical outcomes at baseline and month 12 of the intervention and associated treatment effects in a subset of Auckland participants (*n* = 79) participating in the GUMLi randomised controlled trial (*N* = 160).

	**Intervention Group**						**Intervention effect**	*P*-value^*^	***P*****-value^†^**		
	***n***	**CM**		***n***	**GUMLi**				**Group-by-time**	**Main effect of time**	**Main effect of group**
		**Baseline**	**Month 12**		**Baseline**	**Month 12**					
**Anthropometry**	50			45							
Weight											
kg		10.0 ± 1.3	12.9 ± 1.7		9.9 ± 1.1	12.8 ± 1.3	−0.1 (−0.5, 0.2)	0.394	0.578	<0.005	0.920
*z* score^‡^		0.5 ± 1.1	0.6 ± 1.0		0.5 ± 0.9	0.6 ± 0.9	−0.1 (−0.3, 0.1)	0.467	0.417	0.705	0.811
Length											
cm		75.3 ± 2.9	88.3 ± 3.8		75.7 ± 3.1	88.7 ± 3.6	0.1 (−0.8, 0.7)	0.842	0.982	<0.005	0.253
*z* score^‡^		0.1 ± 1.1	0.4 ± 1.2		0.3 ± 1.1	0.6 ± 1.1	0.0 (−0.3, 0.2)	0.953	0.761	0.001	0.244
BMI											
kg/m^2^		17.5 ± 1.4	16.5 ± 1.5		17.3 ± 1.1	16.2 ± 1.2	−0.2 (−0.6, 0.3)	0.427	0.612	<0.005	0.359
*z* score^‡^		0.6 ± 0.9	0.5 ± 1.0		0.4 ± 0.8	0.2 ± 0.9	−0.1 (−0.4, 0.2)	0.340	0.460	0.011	0.356
WAZ^‡^		−0.12 ± 1.2	0.25 ± 1.1		−0.07 ± 0.9	0.24 ± 1.0	−0.0 (−0.3, 0.2)	0.773	0.720	<0.005	0.772
WLZ^‡^		0.6 ± 0.9	0.5 ± 1.0		0.5 ± 0.8	0.3 ± 0.8	−0.1 (−0.4, 0.1)	0.309	0.377	0.008	0.519
LAZ^‡^		0.14 ± 0.9	0.40 ± 1.1		0.34 ± 1.0	0.54 ± 1.0	0.0 (−0.3, 0.2)	0.713	0.595	0.010	0.284
Body Fat											
%		24.4 ± 6.3	23.5 ± 6.8		22.9 ± 6.7	19.6 ± 6.9	−3.2 (−6.2, −0.3)	0.032	0.180	0.071	0.039
Free Fat Mass											
kg		7.6 ± 1.2	9.9 ± 1.4		7.6 ± 1.1	10.2 ± 1.5	0.2 (−0.2, 0.7)	0.291	0.279	<0.005	0.278
Fat Mass											
kg		2.4 ± 0.7	3.1 ± 1.1		2.3 ± 0.7	2.5 ± 0.9	−0.5 (−0.9, −0.1)	0.028	0.082	0.028	0.039
Fat Mass Index											
kg/m^2^		4.3 ± 1.2	3.9 ± 1.4		4.0 ± 1.2	3.2 ± 1.2	−0.7 (−1.2, −0.1)	0.020	0.170	0.005	0.028
**Macronutrients**	50			45							
Energy											
kcal/d		1336.4 ± 532.2	1539.2 ± 406.7		1254.1 ± 380.1	1637.1 ± 527.1	152.1 (−25.8, 330.1)	0.093	0.086	<0.005	0.605
From CM (kcal/d)		312.6 ± 2061	275.3 ± 97.3		294.1 ± 195.4	257.6 ± 106.5	−12.7 (−54.1, 28.7)	0.544	0.999	0.179	0.579
Protein											
g/d		51.3 ± 18.3	74.2 ± 21.8		50.5 ± 19.4	74.8 ± 27.2	2.3 (−7.5, 12.0)	0.646	0.661	<0.005	0.788
From CM (g/d)		10.3 ± 6.5	15.0 ± 5.6		10.3 ± 7.0	10.3 ± 4.8	−4.6 (−6.7, −2.4)	<0.005	0.005	0.034	0.024
g/1,000 kcal		39.0 ± 7.9	47.3 ± 6.9		39.2 ± 7.1	44.6 ± 6.2	−2.8 (−5.4, −0.1)	0.040	0.146	<0.005	0.197
g/kg/d^2^		5.1 ± 1.7	5.8 ± 1.7		5.1 ± 1.9	6.0 ± 2.4	0.3 (−0.6, 1.1)	0.543	0.524	0.003	0.803
%PI from CM		20.9 ± 13.8	21.7 ± 9.1		21.1 ± 14.6	14.8 ± 7.9	−6.9 (−10.4, −3.4)	<0.005	0.024	0.131	0.086
%EI from CM		3.2 ± 2.0	4.1 ± 1.6		3.1 ± 2.3	2.7 ± 1.4	−1.5 (−2.1, −0.9)	<0.005	0.002	0.797	0.034
**Biochemical**	41			38							
IGF-1^§^											
ng/mL		77.3^§^±58.0	105.5^§^±65.0		71.0^§^±66.1	95.7^§^±43.2	−19.6 (−41.0, 1.9)	0.073	0.698	0.003	0.126
IGFBP-3^§^											
ng/mL		2709.6^§^±759.2	2911.4^§^±821.0		2678.4^§^±991.7	2910.4^§^±714.0	−134.8 (−446.0, 176.4)	0.391	0.874	0.479	0.518

*All data are mean ± SD*.

* *An ANCOVA model was used to test the difference between the two groups, adjusting for baseline outcome, child sex and multiple comparisons (Bonferroni)*.

† *Repeated-measures ANOVA for group (GUMLi compared with CM), with interaction between treatment and time, adjusting for child sex, P < 0.05*.

‡ *The z-score is calculated using World Health Organization child growth standards*.

§ *Geometric mean*.

*CM, Cow's Milk; EI, Energy Intake; GUMLi, Growing Up Milk Lite; IGF-1, insulin-like growth factor I; IGFBP-3, insulin-like growth factor binding protein 3; LAZ, length-for-age z-score; PI, Protein Intake; WAZ, weight-for-age z-score; WLZ, weight-for-length z-score*.

### Anthropometry

Mean weight, length, BMI measurements, their associated *z*-scores and measures of adiposity are summarised in Table 2. No significant group-by-time interaction was seen for any of the anthropometric or growth variables (Supplementary Table 2). Children randomised to receive GUMLi had a 3.2% (95% CI: −6.2, −0.3; *p* = 0.032) lower body fat percentage (BF%) at 2 y compared to children randomised to receive CM, however, no significant group-by-time interaction was seen.

### Serum Biomarkers

Children randomised to receive GUMLi had IGF-1 concentrations 19.6 ng/mL (95% CI:−41.0, 1.9; *p* = 0.0.073) lower than children in the CM group. IGF-1 significantly increased from 1 to 2 years of age (*p* = 0.003) in both groups, however, IGFBP-3 did not change significantly between 1 and 2 years of age and were comparable between groups (Table 2). The milk interventions had no effect on IGF-1 or IGFBP-3 when analysed separately by sex (Supplementary Table 3).

### Correlations

There was no effect of the milk intervention on IGF-1 concentrations at month 12 of the intervention (Table 2). Therefore, the CM and GUMLi groups were combined and associations between IGF-1, IGFBP-3, anthropometry and diet were investigated at baseline and month 12 of the intervention, adjusting for sex (Supplementary Tables 4, 5). At baseline, IGF-1 concentrations were positively correlated with size (zWFL, zBMI, WAZ; all *p* < 0.01, and LAZ, *p* = 0.020). There was a significant positive correlation between IGF-1 and protein per gramme of body weight (*p* = 0.020) and %EI from cow's milk protein (*p* = 0.044). After 12 months of the intervention, IGF-1 at 2 y remained positively correlated with size (zWF, zBMI, and LAZ *p* < 0.05, WAZ *p* < 0.006) after adjustment for sex, breastfeeding status, and gestation. Change in IGF-1 from 12 to 24-months-of-age was significantly positively correlated with weight (zWFL, and zBMI *p* < 0.05 and WAZ, *p* < 0.01). There was a significant association between IGF-1 and total cow's milk intake (*r*_s_ = 0.280, *p* = 0.032) and a trend towards an association between %EI from cow's milk protein (*r*_s_ = 0.245, *p* = 0.061) adjusted for sex, breastfeeding status, and gestation (Supplementary Table 5).

Further, to establish whether associations between IGF-1 at 2 y and protein intake from cow's milk were independent of confounding, a hierarchical multiple linear regression was performed incorporating sex and LAZ at 2 y in the base model (Table 3). LAZ was included in the base model due to its positive bivariate correlation with IGF-1 at 2 y. Body fat percent was not significantly associated with IGF-1 when added into the model (Model 1). In Model 2 we added total protein intake from cow's milk at 2 y and all statistically significant relationships from Model 1 remained, with a significant association between total protein intake from cow's milk and IGF-1. In addition, total protein intake (g/d) from cow's milk at 2 y was positively associated with IGF-1 at 2 y (β = 0.25, 95%CI 0.001, 0.018; *p* = 0.03).

**Table 3:** Standardised beta coefficients from multiple regression predictors of IGF-1 at 2 years of age in a subset of Auckland participants (*n* = 79) participating in the GUMLi randomised controlled trial (*N* = 160).

	**Sex**	**Length-for-age *z*-score**	**Body fat percent at age 2 years**	**Total protein intake (g/d) from CM at age 2 years**
Base model	0.327^†^	0.209		
Model 1	0.325^†^	0.201	−0.031	
Model 2	0.355^†^	0.159	−0.063	0.247^*^

*CM, Cow's Milk; IGF-1, insulin-like growth factor I; IGFBP-3, insulin-like growth factor binding protein 3; PI, Protein Intake*.

* *p < 0.05*;

† *p < 0.01*.

*Base model: child sex, length-for-age at age 2 years*.

*Model 1: child sex, length-for-age at age 2 years, body fat percent at age 2 years*.

*Model 2: child sex, length-for-age at age 2 years, body fat percent at age 2 years, total protein intake from CM*.

## Discussion

In this sub-set of well-nourished, healthy, Auckland-based children from the GUMLi randomised control trial cohort we found no effect of the milk interventions on IGF-1 concentrations at 2 y across the two groups or by sex. As expected, adjusted protein intake (g/1000 kcal), total protein intake from cow's milk-based drinks, and %EI from cow's milk protein decreased significantly in the GUMLi group over the 12-month duration of the trial, with significant group-by-time interactions evident at 2 y. There were no independent effects of the milk intervention at 2 y for anthropometric variables, except BF% and FFM.

Our findings that a higher protein intake from cow's milk in early life is associated with a small increase in adiposity is consistent with observational cohorts (16, 24, 35, 36) and previous randomised controlled trials by Weber et al. (10) and Koletzko et al. (37), which demonstrated a positive causal effect of high protein intakes during infancy on BMI and weight gain in early life. *Post-hoc* analysis revealed a significant decrease in WLZ over the 12-month duration of the study in the GUMLi group only, suggestive of a decreased risk of overweight in the GUMLi group, further supported by the small but significant difference in BF% between groups alongside significant linear growth. These growth patterns are similar to those observed in children randomised into a dairy vs. a meat-based complementary diet from 5 to 12 months of age reported by Tang et al. (20) in their follow up of infants at 2 y. As our study evaluated the effect of two dairy-based protein interventions direct comparisons with meat vs. milk studies is not possible. However, provides some evidence of the potential for a combined effect of the protein-source (even within common animal sources of protein i.e., dairy vs. meat) and the complete food matrix on growth, rather than total protein intake (20, 21, 38). This warrants further research in early childhood.

Globally, current protein intakes in 12–36 months old children are high (39), with a greater risk for increased weight gain, body fat deposition and later risk of obesity, adiposity, and associated comorbidities induced by animal proteins (predominantly dairy protein in the form of cow's milk) compared to plant-based proteins (14). The relationship between protein intake and stimulation of IGF-1 is an important consideration in the context of Growing Up Milks (GUM or YCF), which are frequently given to children in higher-income countries and remains a significant source of energy and protein, with intakes ≤ 500 mL/d providing up to 50% of total protein requirements from 1 y (6, 15, 40, 41). The GUMLi trial study design is unique in that the intervention duration was throughout the second year of life, providing an opportunity to explore the role of diet, particularly the influence of cow's milk as a drink on IGF-1 concentrations and growth beyond 1 y. We hypothesised that the children randomised to receive GUMLi would have lower IGF-1 concentrations, driven by the 45% difference in grammes of protein per 100 mL of the intervention milks. Whilst this analysis contributes to the evidence base exploring the relationship between cow's milk, growth, and IGF-1, it does not afford the ability to infer causality or determine the increased risk associated with exceeding current protein recommendations in the second year of life due to insufficient numbers in each group to detect clinically meaningful differences in outcomes.

Energy-adjusted protein intakes in both groups were higher than previously reported in cross-sectional data of children under 2 y from the US (20, 42), Belgium (5), Ireland (6), and New Zealand (43). This is possibly due to the higher total cow's milk intake in the study (453 mL/d and 431 mL/d, CM and GUMLi groups, respectively) compared to previously reported studies. Differences in the contribution of cow's milk to total protein intake were apparent, with children randomised to the CM group having a 6.9% greater protein intake from cow's milk as a percentage of total daily protein intake compared to the GUMLi group. This difference would equate to a 4.6 g difference in total protein intake from CM, or an additional 150 mL CM per day.

Despite the milk interventions having no independent effects on IGF-1 concentrations, total CM intake (mL/d) at 2 y was independently associated with IGF-1 concentrations at 2 y. The cross-sectional relationship between energy-adjusted protein intake and IGF-1 concentrations in healthy, well-nourished children at 2 y is similar to that reported by Larnkjaer et al. (22), where IGF-1 concentrations were associated with protein intake reported as a percentage of total energy intake at 1 y of age. Providing evidence that total protein intake, particularly protein intake from CM, increases IGF-1 concentrations relative to a child's total energy intake (23, 24, 44). This represents an area of research that warrants further investigation (45).

### Limitations

A limitation of this analysis, is that it is an exploratory analysis of the GUMLi randomised controlled trial which was originally powered to detect a 0.5 SD of difference in body fat percent at 2 y and not differences in IGF-1 concentrations or other anthropometric measurements. Excess frozen serum samples were only available for a subset of the Auckland cohort and not the Brisbane cohort. However, the Auckland cohort encompassed 68% of the GUMLi trial sample. Serum urea nitrogen (SUN), an indicator of total protein intake was not measured due to limited serum sample volume and would have strengthened this analysis as increases in SUN have been reported in 9 and 12-month-old infants randomised to a whole milk intervention, with a trend for difference between milk intervention groups of whole milk and formula (22). Absolute quantities of additional protein sources e.g., from partial breastfeeding were not included in the analyses as this could not be quantified. Both intervention milks delivered the same whey:casein ratio of cow's milk protein, but differed in their delivery of fat (including long chain polyunsaturated fatty acids) and synbiotics. The impact of these compositional differences on fasting insulin was not measured and this should be considered when interpreting these results. Whilst there were no differences between groups in IGF-1 concentrations at 2 y, the >60% difference in total carbohydrates between the CM and GUMLi interventions must be considered, as higher levels of simple carbohydrates such as lactose will have an increased effect on insulin concentrations (44).

## Conclusion

Whilst the reduced protein GUMLi milk intervention in comparison to CM resulted in a small but significant effect on percentage body fat, it had no effect on IGF-1 concentrations at 2 y. Protein intake from CM was positively associated with IGF-1 concentrations on cross-sectional analysis at 2 y. CM remains an important source of nutrition in young children, providing nutrients outside of protein, however, further consideration as to how much it contributes to total protein intakes in early childhood is warranted. The second year of life should be considered as a specific time point where protein-specific dietary interventions could be targeted as children transition to the family diet in an effort to reduce the risk of overweight and adiposity in later childhood.

## Data Availability Statement

The raw data supporting the conclusions of this article will be made available by the authors upon request.

## Ethics Statement

The studies involving human participants were reviewed and approved the Northern B Health and Disability Ethics Committee (HDEC) of the New Zealand Ministry of Health (HDEC reference number 14/NTB/152) and the University of Queensland Medical Research Ethics Committee (MREC) in Brisbane, Australia (reference number 2014001318). Written informed consent to participate in this study was provided by the participants' legal guardian/next of kin.

## Author Contributions

CW, PD, and CG: developed the GUMLi Trial. AL, MM, TM, and RH: conducted the study data. AL and CW: wrote the manuscript. AL: conducted the statistical analyses of the data. All authors have read, contributed, and approved the final manuscript.

## Conflict of Interest

AL has received honoraria for presentations and consultations from Danone Nutricia. CW has received honoraria for presentations and consultations from Danone, Nutricia, Pfizer, and Fonterra. CG has received honoraria for consultations from Fonterra. PD has received honoraria for presentations and consultation from Danone Nutricia, Nestle, Bayer, Diary Australia, H+H, Bellamys, Sanulac, and Aspen Nutritionals. RH is currently employed by Reckitt Benckiser Group, however was employed at the University of Queensland at the time of the GUMLi Trial. The remaining authors declare that the research was conducted in the absence of any commercial or financial relationships that could be construed as a potential conflict of interest.

## Abbreviations

CM: cow's milk
GUM: Growing Up Milk
GUMLi: Growing Up Milk Lite
IGF-1: insulin-like growth factor 1
IGFBP-3: insulin-like growth factor binding protein 3.
