# Supplementary material for: Protein Intake, IGF-1 Concentrations, and Growth in the Second Year of Life in Children Receiving Growing Up Milk – Lite (GUMLi) or Cow's Milk (CM) Intervention
Source: Front Nutr. 2021 Jun 10;8:666228. doi: 10.3389/fnut.2021.666228 (PMC8224403; doi:10.3389/fnut.2021.666228)
Supplement: Supplementary file 1 [file Data_Sheet_1.pdf]

Supplementary Material

Supplementary Table 1: Nutritional composition of CM and GUMLi per 100 mL of prepared product*

	Unit	Study Group	
		CM	GUMLi
Energy	kJ	245.0	249.0
	kcal	58.3	59.3
Protein	g	3.1	1.7†
% of energy	%	21.5	11.6
Carbohydrate	g	4.5	7.8‡
Total fat, g	g	3.1	1.9§
Saturated fat, g	g	1.9	1.3
ALA	g		0.01
DHA	g		0.03
Total omega-3 long chain fatty acids (DHA + EPA + DPA)	g	<0.002	0.04
Dietary fibre			
scGOS	g	0.0	1.8
lcFOS	g	0.0	0.2
Micronutrients			
Non-haem iron	mg	0.0	1.3
Cholecalciferol	µg	0.1	1.2

Abbreviations: CM, Cow's milk; DPA, docosapentaenoic acid; GUMLi, Growing-Up Milk–Lite; lcFOS, long-chain fructooligosaccharides; scGOS, short-chain galacto-oligosaccharides.

* Values obtained from the manufacturer (Danone) and are based on average totals from three batches produced for use in the GUMLi Trial.

† Whey: casein (80:20)

‡ No sucrose, dextrose, or flavours

§ Milk fat + long-chain polyunsaturated fatty acids (LCPUFAs)

Supplementary Table 2: Comparison of outcomes after 12 months intervention with GUMLi (Intervention) or unfortified CM (control).

**Parameter & treatment**	***n***	**Baseline**	**Month 12**	**Paired test***	**Treatment**[†]
**Anthropometry**					
Weight, kg					0.693**
CM	50	10.0 ± 1.3	12.9 ± 1.7	**<0.005**[§]	
GUMLi	45	9.9 ± 1.1	12.8 ± 1.3	**0.003**[§]	
zweight[‡]					0.954**
CM	50	0.5 ± 1.1	0.6 ± 1.1	0.205[§]	
GUMLi	45	0.5 ± 0.9	0.6 ± 0.9	0.489[§]	
Length, cm					0.571**
CM	50	75.3 ± 2.9	88.3 ± 3.8	**<0.005**[§]	
GUMLi	45	75.7 ± 3.1	88.7 ± 3.6	**<0.005**[§]	
zlength[‡]					0.373**
CM	50	0.1 ± 1.1	0.4 ± 1.2	**<0.005**[§]	
GUMLi	45	0.3 ± 1.1	0.6 ± 1.1	**0.001**[§]	
BMI, kg/m^2					0.255**
CM	50	17.5 ± 1.4	16.5 ± 1.5	**<0.005**[§]	
GUMLi	45	17.3 ± 1.3	16.2 ± 1.2	**<0.005**[§]	
zBMI[‡]					0.276**
CM	50	0.6 ± 0.9	0.5 ± 1.0	0.259[§]	
GUMLi	45	0.4 ± 0.8	0.2 ± 0.9	0.081[§]	
WLZ					0.334**
CM	50	0.6 ± 0.9	0.5 ± 1.0	0.224[§]	
GUMLi	45	0.5 ± 0.8	0.3 ± 0.8	**0.028**[§]	
WAZ					0.981
CM	50	-0.1 ±1.2	0.2 ± 1.3	**0.001**	
GUMLi	45	-0.1 ± 0.9	0.2 ± 1.0	**0.004**	
LAZ					0.468
CM	50	0.1 ± 0.9	0.4 ± 1.1	**0.002**	
GUMLi	45	0.3 ± 1.0	0.5 ± 1.0	**0.013**	
Body Fat, %					**0.014****
CM	41	24.4 ± 6.3	23.5 ± 6.8	0.478[§]	
GUMLi	39	22.9 ± 6.7	19.6 ±6.9	**0.002**[§]	
Fat Free Mass, kg					0.349**
CM	41	7.6 ± 1.2	9.9 ± 1.4	**<0.005**[§]	
GUMLi	39	7.6 ± 1.1	10.2 ± 1.5	**<0.005**[§]	
Fat Mass, kg					**0.014**[¶]
CM	41	2.4 ± 0.7	3.1 ± 1.1	**0.002**[‖]	
GUMLi	39	2.3 ± 0.7	2.5 ± 0.9	0.070[‖]	
Fat Mass Index, kg/m^2					**0.024**[¶]
CM	41	4.3 ± 1.2	3.9 ± 1.4	**0.045**[‖]	
GUMLi	39	4.0 ± 1.2	3.2 ± 1.2	**<0.005**[‖]	
**Macronutrients**					
Energy, kJ/d					0.257[¶]
CM	50	5612.8 ± 2235.3	6464.5 ± 1708.3	**0.002**[‖]	
GUMLi	45	5267.2 ± 1596.4	6875.7 ± 2213.9	**<0.005**[‖]	
Protein, g/d					0.976[¶]
CM	50	51.3 ± 18.3	74.2 ± 21.8	**<0.005**[‖]	
GUMLi	45	50.5 ± 19.4	74.8 ± 27.2	**<0.005**[‖]	
Energy from CM, kJ/d					0.876[¶]
CM	50	1312.7 ± 865.7	1156.4 ± 408.6	0.205	
GUMLi n	45	1234.2 ± 830.1	1105.8 ± 421.0	0.333	
Protein from CM, kJ/d					**<0.005**[¶]
CM	50	10.3 ± 6.5	15.0 ± 5.6	**<0.005**	
GUMLi	45	10.3 ± 7.0	10.3 ± 4.8	0.982	
Protein, g/1000kcal					0.085[¶]
CM	50	39.0 ± 7.9	47.3 ± 6.9	**<0.005**	

GUMLi	45	39.2 ± 7.1	44.6 ± 6.2	**<0.005**	
Protein, g/kg/d					0.967[¶]
CM	50	5.1 ± 1.7	5.8 ± 1.7	**0.031**[ǁ]	
GUMLi	45	5.1 ± 1.9	6.0 ± 2.4	**0.045**[ǁ]	
Protein, %PI from CM					**<0.005**[¶]
CM	50	20.9 ± 13.8	21.7 ± 9.1	0.697[ǁ]	
GUMLi	45	21.1 ± 14.6	14.8 ± 7.9	**0.011**[ǁ]	
Protein CM, %EI					**<0.005**[¶]
CM	50	3.2 ± 2.0	4.1 ± 1.6	**0.005**	
GUMLi	45	3.4 ± 2.3	2.7 ± 1.4	0.085	
Serum biomarkers					
IGF-1, ng/mL					0.618[§§]
CM	41	77.3[††] ± 58.0	105.5[††] ± 65.0	**0.001**[‡‡]	
GUMLi	38	71.0[††] ± 66.1	95.7[††] ± 43.2	**<0.005**[‡‡]	
IGFBP-3, ng/mL					0.704[§§]
CM	41	2709.6[††] ± 759.2	2911.4[††] ± 821.0	0.161[‡‡]	
GUMLi	38	2678.4[††] ± 991.7	2919.4[††] ± 714.0	0.061[‡‡]	

Abbreviations: CM, Cow's Milk; GUMLi, ; EI, Energy Intake; Growing Up Milk Lite; IGF-1, insulin-like growth factor I; IGFBP-3, insulin-like growth factor binding protein 3; PI, Protein Intake.

Values are mean ± SD

* Level of significance within groups between baseline and month 12

† Level of significance for difference between groups at month 12

‡ The z-score is calculated using World Health Organization child growth standards

§ Paired-samples t-test

ǁ Wilcoxon signed-rank for non-parametric variables

¶ Mann-Whitney for non-parametric variables

** Independent t-test for parametric variables

†† Geometric mean

‡‡ Paired-samples t-test of log transformed IGF-1 and IGFBP-3

§§ Independent t-test on log transformed IGF-1 and IGFBP-3 data

Supplementary Table 3. IGF-1 and IGFBP-3 concentrations of infants in the milk intervention groups at 1 and 2 y. of age.

**Characteristic**	**Study Group**		**p-value** [b]
	**CM** [a] (*n*= 41)	**GUMLi** [a] (*n*= 38)	
1 y. of age			
IGF-1 (ng/mL)			
All	90.8 ± 58.0 (*41*)	85.2 ± 66.1 (*38*)	0.320
Boys	85.3 ± 53.0 (*28*)	65.3 ± 28.1 (*18*)	0.207
Girls	102.5 ± 68.3 (*13*)	103.2 ± 84.1 (*20*)	0.685
IGFBP-3 (ng/mL)			
All	2802.2 ± 759.2 (*41*)	2822.8 ± 991.8 (*38*)	0.519
Boys	2813.3 ± 823.5 (*28*)	2134.4 ± 909.9 (*18*)	0.113
Girls	2778.3 ± 628.1(*13*)	3035.1 ± 1036.5 (*20*)	0.624
2 y. of age			
IGF-1 (ng/mL)			
All	119.4 ± 65.0 (*41*)	105.4 ± 43.2 (*38*)	0.736
Boys	101.2 ± 45.7 (*28*)	86.4 ± 35.4 (*18*)	0.707
Girls	158.5 ± 83.4 (*13*)	122.5 ± 43.2 (*20*)	0.279
IGFBP-3 (ng/mL)			
All	3012.5 ± 821.0 (*41*)	2997.9 ± 714.0(*38*)	0.739
Boys	2809.2 ± 678.9 (*28*)	2695.5 ± 563.6 (*18*)	0.624
Girls	3450.5 ± 951.5 (*13*)	3270.0 ± 737.6 (*20*)	0.900

Abbreviations: IGF-1, insulin-like growth factor I; IGFBP-3, IGF-binding protein 3

[a] Values are mean ± SD (*n*)

[b] Mann-Whitney for non-parametric variables. Performed separately for boys and girls.

Supplementary Table 4. Partial correlations at baseline between growth, biochemical markers, and dietary intake after controlling for sex.

	zWFL	zBMI	WAZ	LAZ	Body fat, %	Energy, kJ/d	Protein, g/1000 kcal*	Protein, $g.kg.d^{-1}$	Total CM intake, mL/d	Total CM, %PI	Total protein from CM, %EI	IGF-1	IGFBP-3
WLZ	1.000	0.974‡	0.877‡	0.418†	0.073	0.228†	0.079	-0.017	0.153	0.050	0.109	0.307‡§	0.282‡
zBMI		1.000	0.754‡	0.211†	0.123	0.183	0.061	-0.037	0.116	0.049	0.101	0.269‡§	0.057
WAZ			1.00	0.788‡	-0.033	0.291‡	0.084	0.030	0.216†	0.066	0.125	0.338‡§	0.385‡
LAZ				1.00	-0.170	0.257†	0.081	0.071	0.216†	0.050	0.089	0.243†§	0.388‡
Body Fat, %					1.000	-.265†	-0.017	-0.272‡	-0.240†	-0.104	-0.098	-0.170§	-0.128
Energy, kJ/d						1.000	-0.149	0.765‡	0.303‡	-0.032	-0.047	0.184§	0.304‡
Protein, g/1000kcal*							1.000	0.391‡	-0.103	-0.207†	-0.027	0.199§	0.059
Protein, $g.kg.d^{-1}$								1.000	0.214†	-0.128	-0.048	0.244†§	0.194
Total CM Intake, mL/d									1.000	0.844‡	0.843‡	0.145§	0.218†
Total CM, %PI										1.000	0.966‡	0.131§	0.124
Total protein from CM, %EI											1.000	0.212†§	0.163
IGF-1												1.000	0.709‡
IGFPB-3													1.000

Abbreviations: CM, Cow's Milk; IGF-1, insulin-like growth factor I; IGFBP-3, insulin-like growth factor binding protein 3; %EI, Percent Energy Intake; %PI, Percent protein intake; PI, Protein Intake.
* Energy-adjusted per 1000 kcal (density method)
† P<0.05
‡ P<0.01
§ adjusted for still breastfeeding and gestation (>37 weeks, <37 weeks)

Supplementary Table 5. Partial correlations at month 12 between growth, biochemical markers, and dietary intake after controlling for sex.

	zWFL	zBMI	WAZ	LAZ	Body fat, %	Energy, kJ/d	Protein, g/1000 kcal*	Protein, $g.kg.d^{-1}$	Total CM intake, mL/d	Total CM, %PI	Total protein from CM, %EI	IGF-1	ΔIGF-1	IGFBP-3	ΔIGFBP-3
WLZ	1.000	0.992‡	0.822‡	0.290†	0.237	-0.062	0.052	-0.334†	0.132	0.099	0.109	0.271†§	0.312†	0.323†	0.148
zBMI		1.000	0.754‡	0.173	0.283†	-0.079	0.053	-0.323†	0.093	0.083	0.096	0.263†§	0.309†	0.254†	0.152
WAZ			1.000	0.727‡	-0.010	-0.005	0.073	-0.319†	0.220	0.125	0.131	0.293†§	0.237†	0.364‡	0.070
LAZ				1.000	-0.267	0.153	0.007	-0.136	0.297†	0.109	0.090	0.260†§	0.046	0.357‡	-0.067
Body Fat, %					1.000	0.051	0.173	0.099	0.041	0.037	0.114	0.036§	-0.047	-0.107	0.002
Energy, kJ/d						1.000	0.113	0.826‡	0.305†	-0.428‡	-0.374‡	0.025§	0.043	0.132	0.129
Protein, g/1000kcal[a]							1.000	0.486‡	0.115	-0.166	0.109	0.168§	0.198	0.119	0.159
Protein, $g.kg.d^{-1}$								1.000	0.192	-0.448‡	-0.301†	0.005§	0.005	0.015	0.139
Total CM Intake, mL/d									1.000	0.583‡	0.653‡	0.280†§	-0.068	0.423‡	-0.081
Total CM, %PI										1.000	0.949‡	0.185§	-0.195	0.306†	-0.204
Total protein from CM, %EI											1.000	0.245§	-0.168	0.325†	-0.169
IGF-1												1.000	0.111	0.694‡	0.004
ΔIGF-1													1.000	-0.298†	0.688‡
IGFPB-3														1.000	-0.066
ΔIGFBP-3															1.000

Abbreviations: CM, Cow's Milk; IGF-1, insulin-like growth factor I; IGFBP-3, insulin-like growth factor binding protein 3; ; LAZ, length –for-age z-score; %EI, Percent Energy Intake; %PI, Percent protein intake; PI, Protein Intake; WAZ, weight-for-age z-score; zBMI, body mass index z-score; zWFL, weight-for-length z-score; ΔIGF-1, change in IGF-1 (baseline – month 12); ΔIGFBP-3, change in IGFBP-3 (baseline – month 12)

* Energy-adjusted per 1000 kcal (density method)

† P<0.05

‡ P<0.01

§ adjusted for still breastfeeding and gestation (>37 weeks, <37 weeks)
